# Antitumor, antioxidant and anti-inflammatory activities of kaempferol and its corresponding glycosides and the enzymatic preparation of kaempferol

**DOI:** 10.1371/journal.pone.0197563

**Published:** 2018-05-17

**Authors:** Jingqiu Wang, Xianying Fang, Lin Ge, Fuliang Cao, Linguo Zhao, Zhenzhong Wang, Wei Xiao

**Affiliations:** 1 Co-Innovation Center for Sustainable Forestry in Southern China, Nanjing Forestry University, Nanjing, China; 2 College of Chemical Engineering, Nanjing Forestry University, Nanjing, China; 3 Jiangsu Key Lab for the Chemistry & Utilization of Agricultural and Forest Biomass, Nanjing, China; 4 Jiangsu Kanion Pharmaceutical Co., Ltd., Lianyungang, China; Duke University School of Medicine, UNITED STATES

## Abstract

Kaempferol (kae) and its glycosides are widely distributed in nature and show multiple bioactivities, yet few reports have compared them. In this paper, we report the antitumor, antioxidant and anti-inflammatory activity differences of kae, kae-7-O-glucoside (kae-7-O-glu), kae-3-O-rhamnoside (kae-3-O-rha) and kae-3-O-rutinoside (kae-3-O-rut). Kae showed the highest antiproliferation effect on the human hepatoma cell line HepG2, mouse colon cancer cell line CT26 and mouse melanoma cell line B16F1. Kae also significantly inhibited AKT phosphorylation and cleaved caspase-9, caspase-7, caspase-3 and PARP in HepG2 cells. A kae-induced increase in DPPH and ABTS radical scavenging activity, inhibition of concanavalin A (Con A)-induced activation of T cell proliferation and NO or ROS production in LPS-induced RAW 264.7 macrophage cells were also seen. Kae glycosides were used to produce kae via environment-friendly enzymatic hydrolysis. Kae-7-O-glu and kae-3-O-rut were hydrolyzed to kae by β-glucosidase and/or α-L-rhamnosidase. This paper demonstrates the application of enzymatic catalysis to obtain highly biologically active kae. This work provides a novel and efficient preparation of high-value flavone-related products.

## Introduction

Kae is a major flavonoid aglycone extract from the *Zingiberaceae Kaempferia* rhizome and mainly exists in nature as the glycoside form [1]. Many beneficial functions of kae and its glycosides have been reported, such as cardiovascular [2], antioxidant [3], antidiabetic [4], anti-inflammatory [5], hepatoprotective [6] and neuroprotective effects [7, 8]. Kae is gaining attention due to its applications in cancer chemotherapy and its other various pharmacological effects [9, 10]. Epidemiological evidence suggests that the consumption of kae-rich foods may reduce the risk of developing some types of cancer, including liver cancer, colon cancer and skin cancer [11, 12]. Inhibiting cancer proliferation and promoting cancer cell apoptosis are the main chemical mechanisms for cancer prevention [13, 14]. Protein kinase B (PKB), also known as AKT, plays an important role in cell survival and apoptosis. Inhibition of PI3K and de-phosphorylation of Akt at Ser473 and Thr308 were observed in K562 and U937 cells after kae treatment [15]. Caspases are a family of cysteine proteases involved in the initiation and execution of apoptosis. Kae has been found to induce the activation of caspase-3, caspase-7, caspase-9 and PARP [16]. In addition, accumulating evidence suggests that reactive oxygen species (ROS) have an important role in cancer development [17]. ROS are byproducts of aerobic metabolism, such as oxygen ions, superoxide anions, peroxides, hydroxyl radicals, oxygen free radicals, and nitric oxide (NO). They play a key role in carcinogenesis, as indicated by increased ROS in cancer cells, ROS-induced malignant cell transformation, and reduced ROS levels leading to malignant cancer cell phenotype reversal [18]. Numerous reports have shown that kae, some kae glycosides, and several kae-containing plants can decrease superoxide anion, hydroxyl radical and peroxynitrite levels [19]. Inflammation has also been suggested to have a significant role in cancer [20]. Inflammatory cells, chemokines and cytokines are present in all tumor microenvironments studied in experimental animal models and humans from the earliest stages of development. Both in vitro and in vivo anti-inflammatory activity have been reported for kae, kae glycosides and/or kae-containing plants [21]. The anti-inflammatory activity of kae may be mediated by several mechanisms of action. Kae can inhibit LPS- and ATP-induced phosphorylation of PI3K and AKT in cardiac fibroblasts, thereby protecting cells from inflammatory injury [22]. Kae and some of its glycosides can also significantly inhibit the production of NO and tumor necrosis factor-alpha (TNF-α) in RAW 264.7 cells stimulated by LPS [23]. Although significant research has focused on the activity of kae aglycone and its glycosides, few studies have compared their activities.

Due to the low concentration of kae aglycone and high concentrations of kae glycosides in plants [24, 25], deglycosylation may provide a way to produce kae from kae glycosides. Modification of flavonoids via glycosylation can be achieved using chemical or biological methods. Compared to chemical methods, biological methods have attracted attention due to their ability to catalyze hydrolysis reactions under milder conditions yielding highly stereo- and regioselective products. At present, common biological methods include enzyme- and microbe-induced transformations. Enzymes have received the most attention due to their many advantages, such as strong selectivity, mild reaction conditions, easy separation and purification, and environmental friendliness. The enzymatic hydrolysis of flavone glycosides to prepare flavone aglycones has been explored using β-glucosidase and α-L-rhamnosidase.

In this study, we investigated the antitumor, antioxidant and anti-inflammatory activities of kae, kae-7-O-glu, kae-3-O-rha and kae-3-O-rut. We demonstrated that kae has better antitumor activity, possibly because kae significantly inhibits AKT phosphorylation and caspase-3, caspase-7, caspase-9 and PARP cleavage while the other kae glucosides do not. Kae also demonstrated better antioxidant and anti-inflammatory activities. To explore and optimize kae glucoside hydrolysis, α-L-rhamnosidase and β-glucosidase were chosen due to their selectivity and previous use in our laboratory. Only β-glucosidase could hydrolyze kae-7-O-glu to kae, but both β-glucosidase and α-L-rhamnosidase could hydrolyze kae-3-O-rut to kae. After optimizing the reaction conditions, complete hydrolysis was achieved with both enzymes.

## Materials and methods

### Chemicals and reagents

Roswell Park Memorial Institute-1640 medium (RPMI-1640), Dulbecco’s modified Eagle’s medium (DMEM) and fetal bovine serum (FBS) were purchased from HyClone (USA). Sodium chloride (NaCl), potassium chloride (KCl), potassium hydrogen phosphate trihydrate (K_2_HPO_4_·3H_2_O), and disodium hydrogen phosphate anhydrous (Na_2_HPO_4_) were purchased from Nanjing Chemical Reagent Co., Ltd. (Nanjing, China). Concanavalin A (Con A), MTT, and propidium iodide (PI) were purchased from Sigma (USA). Trypsin digestion solution, the annexin V-FITC apoptosis detection kit, and WB and IP cell lysates were purchased from Beyotime Biotechnology (Shanghai, China). Kae, rutin, kae-7-O-glu, kae -3-O-rha and kae-3-O-rut standards were purchased from Nanjing Jing Zhu Biotechnology Co., Ltd. (Nanjing, China). The BCATM protein assay kit was purchased from Pierce (USA). N,N'-Methylenebisacrylamide (Bis), sodium dodecyl sulfate (SDS), tris(hydroxymethyl)aminomethane (Tris) and glycine were purchased from Amresco (USA). Ammonium persulfate (APS) was purchased from Nanjing Sunshine Biotechnology Co. Ltd. (Nanjing, China). N,N,N,N'-Tetramethylethylenediamine (TEMED) was purchased from Wako Pure Chemical Industries, Ltd. (Japan). A prestained protein ladder was purchased from Thermo Scientific (USA). Polyvinylidene fluoride (PVDF) was purchased from Millipore (USA). The following antibodies were purchased: anti-cPARP, anti-GAPDH, anti-cleaved-caspase-3, anti-cleaved-caspase-7, anti-cleaved-caspase-9, and anti-p-AKT. 20X LumiGLO Reagent and 20X Peroxide was purchased from Cell Signaling Technology (USA). HRP-coupled anti-mouse and anti-rabbit IgG secondary antibodies and substrates were purchased from KPL (USA). All reagents were of the highest purity commercially available.

### Cell culture

Human hepatoma cell line HepG2, mouse colon cancer cell line CT26, mouse melanoma cell line B16F1 and mouse peritoneal macrophage RAW 264.7 were purchased from the Cell Bank of the Chinese Academy of Sciences. HepG2, CT26 and B16F1 cells were grown in DMEM medium supplemented with 10% fetal bovine serum. RAW 264.7 cells were grown in RPMI-1640 medium supplemented with 10% fetal bovine serum. All cells were kept in a humidified incubator with 5% CO_2_ and 95% air at 37°C. Cells were routinely collected by centrifugation at 800 g for five minutes.

### Recombinant glycosidases

The following recombinant enzymes were previously generated in our laboratory: glucosidase derived from *Thermotoga petrophila DSM 13995* [26] and rhamnosidase derived from *Aspergillus terreus* [27].

### Assessment of cell proliferation with the MTT assay

After trypan blue staining, 3 x 10^3^ tumor cells were inoculated into each well of a 96-well plate. After 6 h, various concentrations of drugs were added to each well, and then, the plate was cultured at 37°C in a 5% CO_2_ atmosphere for 72 h. MTT solution (20 μl, 4 mg/ml) was added to the culture medium. After 4 h, the plate was centrifuged at 1000 g for 5 min, and the supernatant was removed. DMSO (200 μl) was added to the wells and mixed until the precipitate completely dissolved. The absorbance at 540 nm was measured and recorded. All experiments were performed in at least four parallels and repeated three times. The percentage of cell proliferation inhibition was calculated using the following formula:

$$\text{Inhibition}\;\text{rate}\;\text{of}\;\text{cell}\;\text{proliferation}\;(\%)=\left\{1-\frac{{\text{OD}}_{\text{sample}}}{{\text{OD}}_{\text{control}}}\right\}\times 100\%$$

### Apoptosis assay

Cell solutions (1×10^6^ cells) were transferred into a flow cell tube at 4°C and centrifuged at 1000 g for 5 min. The supernatant was removed, and 200 μl of binding buffer (10 mM HEPES, pH 7.4, 140 mM NaCl, 2.5 mM CaCl_2_) was added. After mixing, 1.25 μl of annexin V solution (1 μg/ml) and 2 μl of PI solution (100 μg/ml PI, HEPES 20 mM, pH 7.4) were added. Following lucifugal incubation at room temperature for 20 min, binding buffer (300 μl) was added. The fluorescence in the FL-1 and FL-3 channels was measured by flow cytometry. Data were collected using CellQuest software (BD Immunocytometry Systems) and analyzed using FlowJo software. All experiments were repeated three times.

### Western blotting analysis

The cells were lysed using 150 μl of lysate buffer (10 mM HEPES, 2 mM EDTA, 0.1% CHAPS, 5 mM DTT and 1 mM PMSF). After being chilled in an ice bath for 0.5 h, cells were centrifuged at 12000 g for 2 min at 4°C. The supernatant was removed and the protein concentration was measured using the BCATM protein quantification kit. The protein content of each 50 g sample was electrophoresed in a 10% SDS-PAGE gel at a constant 20 mA current for 1 h at low temperature (4°C). After electrophoresis, the proteins were transferred onto a PVDF membrane by wet rotation. The membranes were incubated with blocking buffer (2% free fat milk, 10 mM Tris-Cl, 50 mM NaCl 0.1% Tween 20, pH 7.4) at room temperature for 2 h to saturate the PVDF membrane. The membrane was cut according to the indicated position of the pre-dyed protein ladder and incubated with primary antibody overnight on the converter. The next day, the membrane was washed in the washing buffer (10 mM Tris-Cl, 50 mM NaCl, 0.1% Tween 20, pH 7.4) three times, twice for 5 min and once for 10 min, to remove any nonspecific primary antibody binding. Next, the membrane was incubated with an appropriate dilution of secondary antibody at room temperature for 2 h. The substrate was diluted (1:20) and incubated with the membrane for 1 minute (ECL). A photograph of the gel was taken, and the relative band density was analyzed by optical densitometry using ImageJ. All experiments were repeated three times.

### Assay for DPPH and ABTS radical scavenging

The DPPH scavenging capacity was measured as previously described [28] with slight modifications. In brief, the sample (100 μl) was added into 100 μl of DPPH solution (0.5 mM DDPH solution diluted in 95% ethanol) and incubated in a 96-well plate at room temperature for 30 min. The sample absorbance (A), ethanol absorbance (B) or control absorbance (C) were measured at 517 nm. The DPPH scavenging capacity was calculated using the following formula:

$$\text{Scavenging}\;\text{capacity}\;(\%)=\left(1-\frac{A-B}{C}\right)\;\text{x}\;100\%$$

The ABTS kit instructions were followed for the ABTS assay (Beyotime, China) [29]. Briefly, ABTS solution (200 μl) and sample (10 μl) were added to each well of a 96-well plate. The plate was gently mixed and incubated at room temperature for 5 min. The sample absorbance (A), ethanol absorbance (B) and control absorbance (C) were measured at 734 nm. The ABTS scavenging capacity was calculated using the above formula. All experiments were performed in at least four parallels and repeated three times.

### Lymphocyte transformation test

The lymphocyte suspension was diluted to 1 x 10^7^ cells/ml. Cell solutions (1 x 10^6^ cells) and Con A (2.5 μg/ml) were added to each well at different concentrations, and the plate was placed in the CO_2_ incubator at 37°C for 48 h. Then, MTT (20 μl) was added, and cells were grown for 4 h. The plate was centrifuged at 1000 g for 5 min; then, the supernatant was removed, and 200 μl DMSO was added to each well. The OD values at 540 nm were measured and recorded. All experiments were performed in at least four parallels and repeated three times.

### Assay for NO production and intracellular reactive oxygen species

RAW 264.7 cells were grown to a density of 2 x 10^5^ cells/ml in the logarithmic phase. The cells (100 μl) were then added to each well of a 96-well plate. After 24 h, the cells were treated with LPS (500 ng/ml) and various concentrations of kae, kae-7-O-glu, kae-3-O-rha and kae-3-O-rut. Each concentration was tested twice in triplicate with the control being the culture medium containing DMSO. Each sample (100 μl) was added to an enzyme-labeled plate and the mixed Griess reagent (100 μl) was added. After 10 min, the absorbance was measured at 540 nm. The inhibitory effect of NO release was calculated using the following formula:

$$\text{NO}\;\text{inhibition}\;\text{rate}\;(\%)=\frac{{\text{OD}}_{\text{LPS}}-{\text{OD}}_{\text{LPS}+\text{sample}}}{{\text{OD}}_{\text{LPS}}-{\text{OD}}_{\text{blank}}}\times 100\%$$

Intracellular ROS were determined as as previously described [30] with slight modifications by using the Reactive Oxygen Species Assay Kit (Beyotime Biotechnology, China). All experiments were performed in at least four parallels and repeated three times.

### Expression of recombinant protein in Escherichia coli

The genetically engineered recombinant strain was cultured on an LB + Amp plate at 37°C for one day. A single colony was isolated from the plate, inoculated in LB + Amp liquid media (4 ml), and shaken at 180 r/min for 8 h at 37°C. The sample was then inoculated in LB + Amp liquid media (150 ml) and shaken again at 180 r/min for 10 h at 37°C. Bacteria were collected via centrifugation at 8000 rpm.

### Enzyme activity assay

After mixing and preheating at 35°C for 10 min, the diluted enzyme solution (10 μl) was added to the buffer solution (150 μl, 100 mM, pH 6.5) with pNPR/pNPG artificial substrate (40 μl, 5 mM). Then, a Na_2_CO_3_ solution (600 μl, 1 M) was added to terminate the reaction, and the absorbance was measured at 405 nm. Enzyme solutions that inactivated the substrate were compared. All experiments were repeated three times.

### Enzymatic hydrolysis of kae-3-O-rut and kae-7-O-glu with α-L-rhamnoside and/or β-glycosidase

All enzymatic reactions were carried out in a temperature-controlled, heated water bath. In this study, a disodium hydrogen phosphate-citrate buffer (pH 3.5–6.5) was used. The typical reaction mixture (200 μl) contained disodium hydrogen phosphate-citrate buffer, substrate (2 mM) and distilled water. The reaction was initiated by combining buffered enzyme solutions. The mixtures were incubated at various pH values, temperatures, enzyme concentrations and times with other conditions remaining fixed. The reaction was stopped by adding 800 μl of methanol. The crude hydrolysis products were then centrifuged at 10000 rpm for 10 min, and the supernatant solutions were filtered through a 0.45 μm filter before injection into the HPLC. All experiments were repeated three times.

## Results

### Evaluation of cytotoxicity against HepG2, CT26 and B16F1 cells

**Fig 1:**
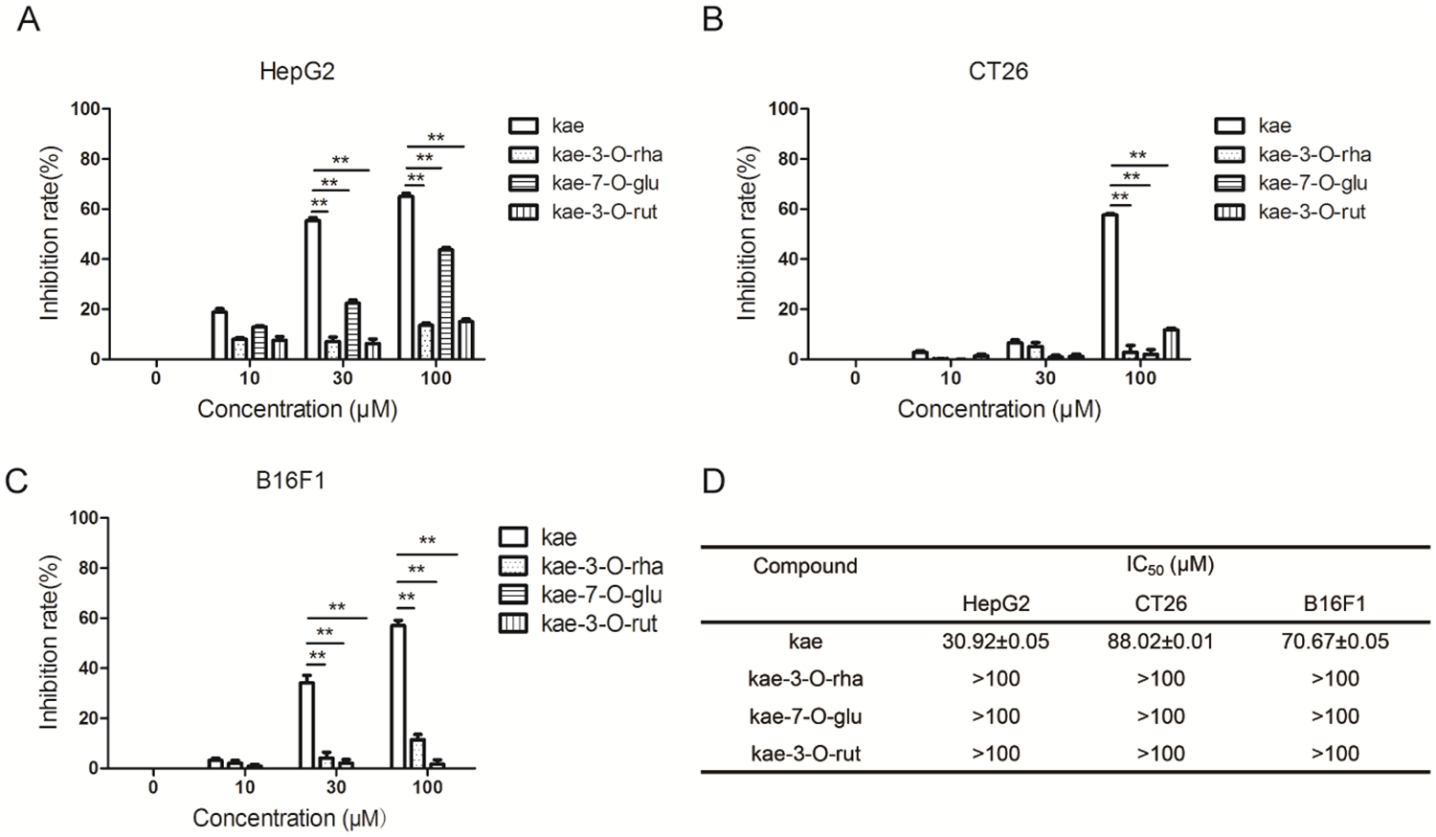
Kae exhibits greater antitumor effects than its glycosides. Cells (3 x 10^3^/well) were added to wells in a 96-well plate and incubated with various concentrations of kae, kae-3-O-rha, kae-7-O-glu and kae-3-O-rut for 72 h. The inhibitory rate of kae, kae-7-O-glu, kae-3-O-rha and kae-7-O-rut on cell proliferation was determined by the MTT assay. (A) HepG2 cells. (B) CT26 cells. (C) B16F1 cells. (D) IC_50_ values of kae, kae-3-O-rha, kae-7-O-glu and kae-3-O-rut. *P < 0.05, **P < 0.01. The data are shown as the mean ± SEM of three independent experiments.

In this paper, kae, kae-3-O-rha, kae-7-O-glu and kae-3-O-rut were evaluated for their antiproliferative activities against HepG2, CT26 and B16F1 cells using the MTT assay. Kae-3-O-rha and kae-3-O-rut had no effects on human hepatocellular carcinoma cell line HepG2 proliferation, while kae and kae-7-O-glu (10–100 μM) caused decreased proliferation with kae showing the strongest inhibitory effects (Fig 1). In both human colon cancer cells CT26 and mouse melanoma cells B16F1, only kae had an inhibitory effect, while the other three compounds had no effect.

### Effect on HepG2 cell apoptosis

**Fig 2:**
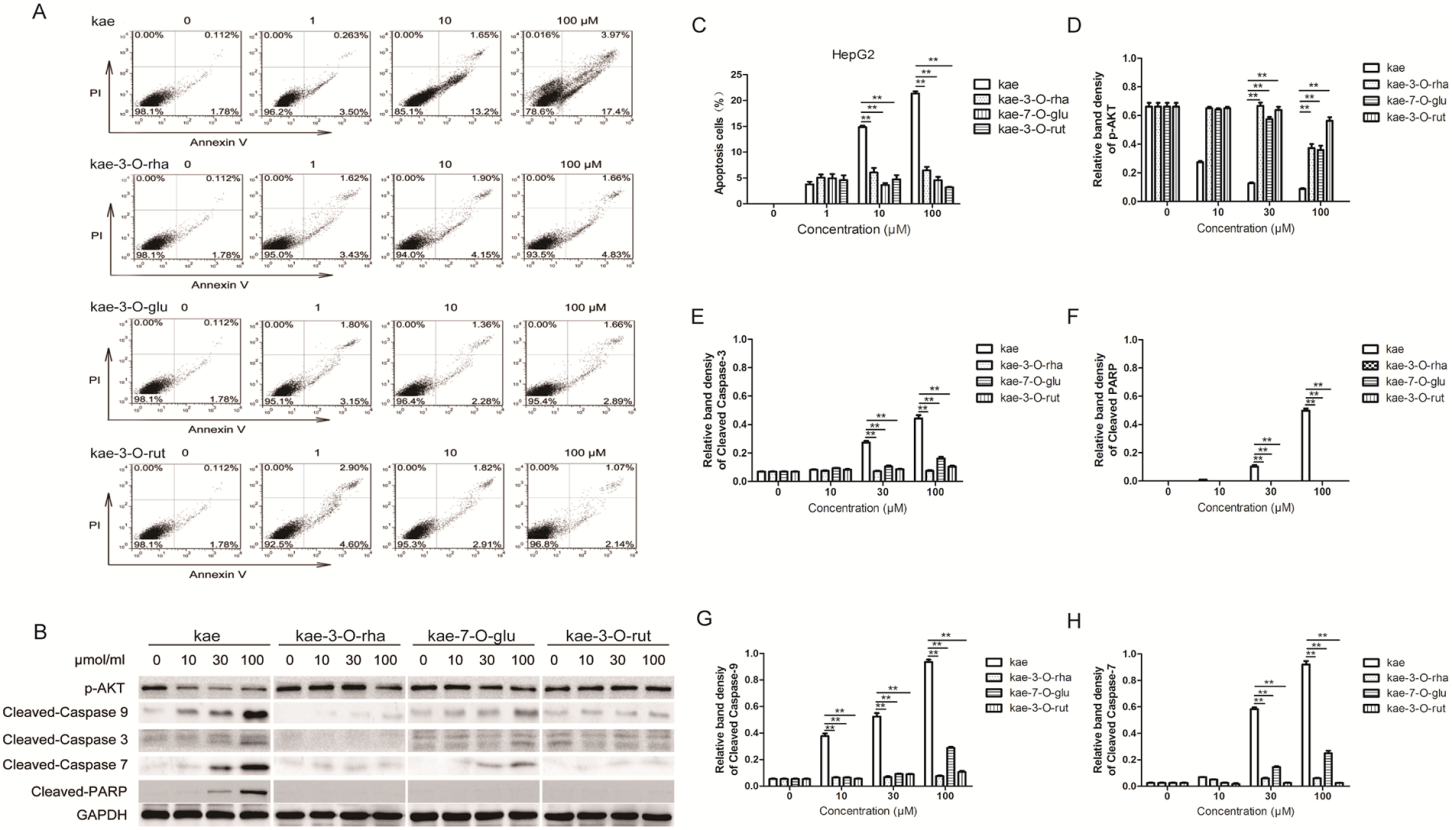
Kae induces liver cancer cell apoptosis, inhibits AKT phosphorylation, and induces caspase-dependent apoptosis in liver cancer cells. (A) HepG2 cells were added to a 96 -well plate and incubated with kae, kae-3-O-rha, kae-7-O-glu and kae-3-O-rut (0, 10 or 100 μM) for 24 h. HepG2 cell apoptosis was determined by annexin V/PI staining. (B) HepG2 cells were treated with kae, kae-3-O-rha, kae-7-O-glu and kae-3-O-rut (10, 30 or 100 μM) for 24 h. Cleaved caspase-3, 7, and 9, cleaved PARP, and p-AKT protein levels were determined by Western blotting. (C) HepG2 cell apoptosis of three independent experiments were shown in the bar graph. (D)-(H) ImageJ software was used to analyze the levels of p-AKT, cleaved caspase-3, 7, 9 and cleaved PARP with GAPDH as the reference. *P < 0.05, **P < 0.01 The data are shown as the mean ± SEM of three independent experiments.

The ability of various compounds to promote HepG2 cell apoptosis was assessed by flow cytometry. Increasing kae concentrations (0–100 μM) caused increasing apoptosis rates (1.78–17.4%), indicating a proportional relationship between kae concentration and tumor cell apoptosis (Fig 2). However, kae-3-O-rha, kae-7-O-glu and kae-3-O-rut (0–100 μM) showed no significant effects on tumor cell apoptosis. To explore the differences in their apoptosis-promoting abilities, we examined the effects of these four compounds on the expression of related proteins in the apoptotic pathway using Western blotting. The expression of cell apoptosis marker proteins, such as cleaved-PARP, cleaved caspase-3, cleaved caspase-7 and cleaved caspase-9, were upregulated in kae-treated cells. This upregulation can significantly inhibit the phosphorylation of the proliferation signal AKT. However, the other three compounds had no significant effects on apoptotic protein expression.

Caspase-3, a cysteine protease, is the key protease in apoptosis. Caspases can transmit apoptotic signals or directly act as apoptotic effector molecules causing apoptosis changes, such as chromatin condensation and DNA fragmentation [31, 32]. Normally, caspase-3 is an inactive zymogen that exists in the cytoplasm. Stimulated by the apoptotic signal, caspase-3 is activated by protease hydrolysis to become four dimers. The activated caspase-3 cleaves molecules in the cytoplasm and cell nucleus substrates, eventually leading to cell apoptosis.

### Antioxidant activity differences

**Fig 3:**
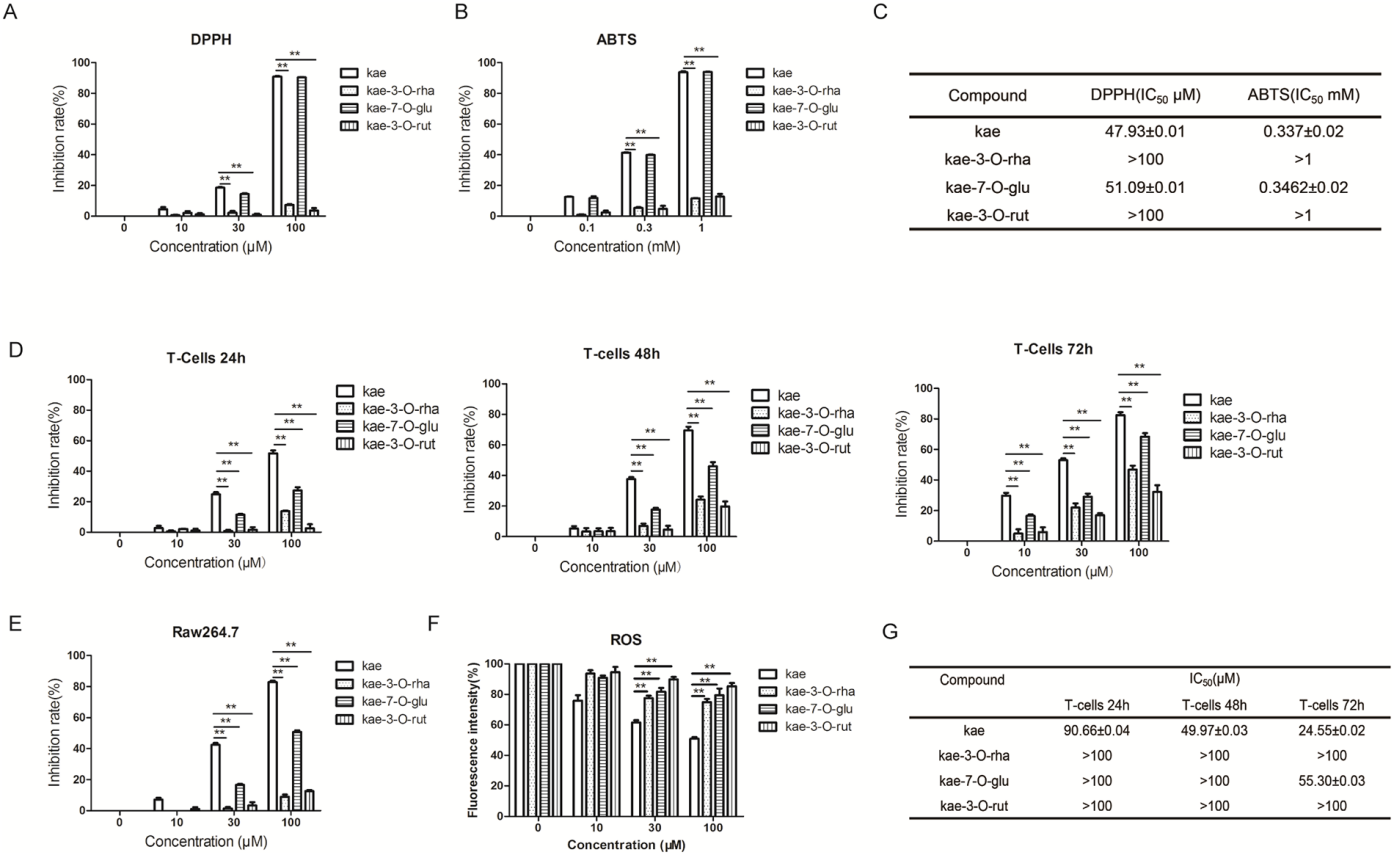
Increased antioxidant and anti-inflammatory effects of kae compared to kae glycosides. (A) The DPPH radical scavenging assay. (B) The ABTS radical scavenging assay. (C) IC_50_ values for DPPH and ABTS radical scavenging. (D) The T cells (1 x 10^6^/well) were added to a 96-well plate with ConA (2.5 g/ml). Various concentrations of kae, kae-7-O-glu, kae-3-O-rha and kae-3-O-rut (0, 10, 30 or 100 μM) co-cultured with activated T cells were incubated for 24 h, 48 h and 72 h. The MTT assay was used to detect ConA-activated T cell proliferation. (E) RAW 264.7 cells (2 x 10^5^ cells/ml) were added to a 96-well plate (100 μl/well). After 24 h, the cells were treated with LPS (500 ng/ml) and kae, kae-7-O-glu, kae-3-O-rha or kae-3-O-rut at various concentrations. Each concentration was tested twice in triplicate with the culture medium containing DMSO as the control. Each sample (100 μl) was added to an enzyme-labeled plate and the mixed Griess reagent (100 μl) was added. After 10 min, the absorbance was measured at 540 nm. The inhibitory effect of NO release on RAW 264.7 cells was calculated. (F) Steady state ROS concentration in the culturing medium was determined using the Reactive Oxygen Species Assay Kit (Beyotime Biotechnology, China). (G) IC_50_ values for the inhibition of T cells. *P < 0.05, **P < 0.01. The data are shown as the mean ± SEM of three independent experiments.

DPPH and ABTS methods were used to compare the antioxidant activities of the four compounds. Kae showed the strongest free radical scavenging capacity, followed by kea-7-glu, and weaker activity from the other two compounds (Fig 3A–3C). Therefore, kae had the strongest antioxidant activity.

### Anti-inflammatory activity differences

First, we examined the effects of the four compounds on ConA-activated T cell proliferation [33]. Kae (100 μM) showed inhibitory effects on activated T cell proliferation, with an inhibitory rate of 53.62% after 24 h, 86.7% after 48 h, and background-level cell suppression at 72 h. The most active kae glycoside, kae-7-O-glu, showed inhibition rates up to 25.5% after 24 h, 51.12% after 48 h and up to 72.05% after 72 h. In general, these four compounds inhibited the proliferation of activated T cells in a time- and dose-dependent manner, with kae exerting the strongest activity. Current research has demonstrated the participation of reactive oxygen species in inflammation. Stimulation of LPS-induced RAW 264.7 cells [34, 35] led to overproduction of NO. Kae and kae-7-O-glu significantly inhibited NO release, while the other two compounds had no significant effect (Fig 3E). Besides, kae inhibited LPS induced ROS production in a concentration-dependent manner, while the other three compounds had no significant effect (Fig 3F).

### Enzymatic hydrolysis of kae-3-O-rha, kae-7-O-glu and kae-3-O-rut

**Fig 4:**
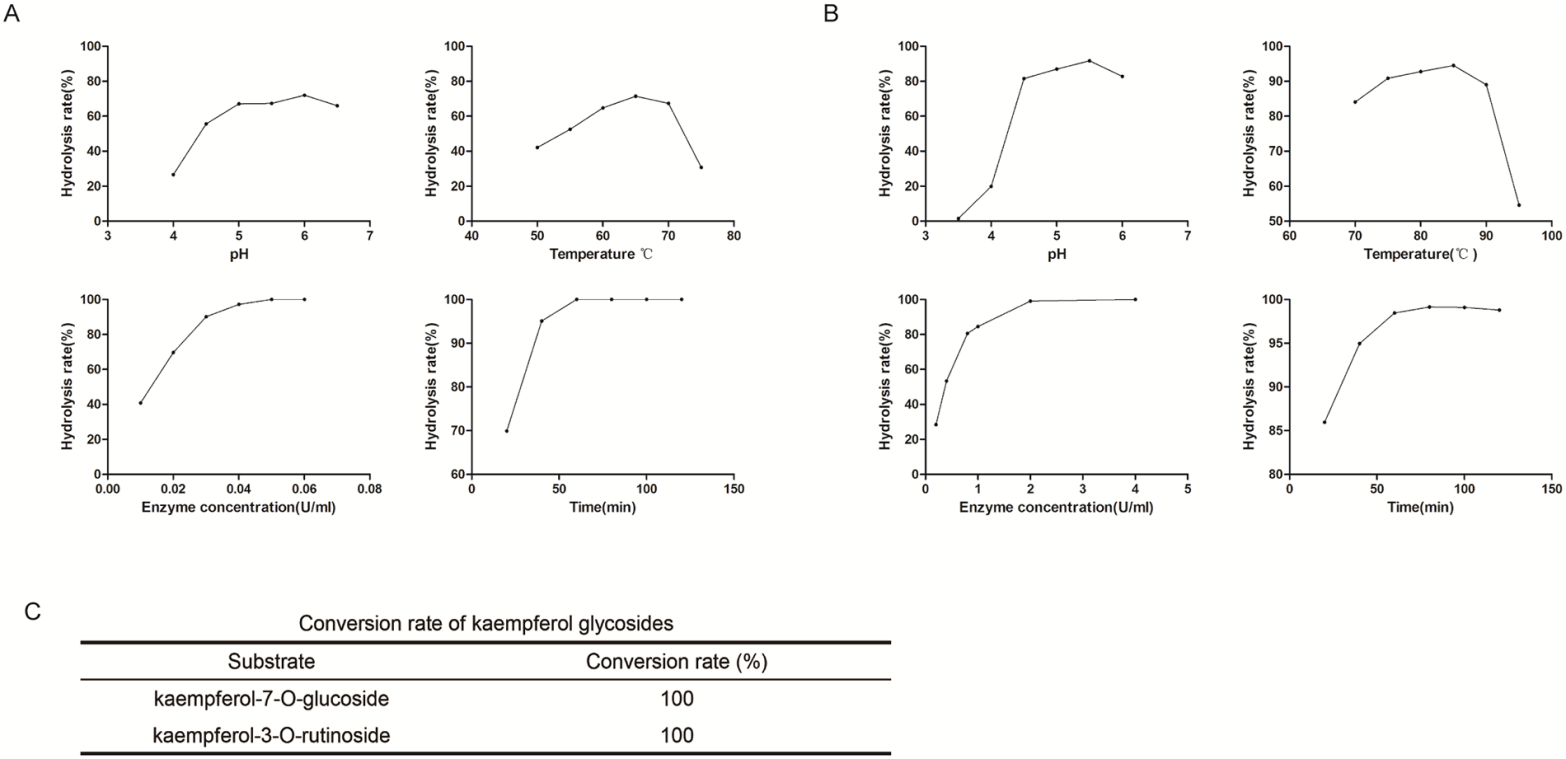
Hydrolysis of kae glycosides by α-L-rhamnoside and/or β-glycosidase. Effects of pH value, temperature, enzyme concentration and time on the hydrolysis rate of kae-3-O-rut by α-L-rhamnoside (A) and kae-3-O-glu by β-glucosidase (B). (C) Hydrolysis rates of kae-3-O-rut and kae-7-O-glu by α-L-rhamnoside and/or β-glucosidase under the optimal conditions. Each value represents the mean of three independent measurements.

First, we chose to study the hydrolytic effects of α-L-rhamnoside and β-glycosidase, which were used in previous studies. Kae-3-O-rut and kae-7-O-glu can be hydrolyzed to kae by α-L-rhamnoside and/or β-glucosidase, while kae-3-O-rha could not be hydrolyzed. To improve the hydrolysis rates of kae-3-O-rut and kae-7-O-glu, various pH values, temperatures, enzyme concentrations and reaction times were examined. The optimal kae-3-O-rut hydrolysis conditions were 0.05 U/ml of α-L-rhamnoside at pH 6.0 and 65°C for 60 min (Fig 4). As α-L-rhamnoside only could hydrolyze kae-3-O-rut to kae-3-O-glu, we next used β-glycosidase to hydrolyze kae-3-O-glu to kae. The optimal kae-3-O-glu hydrolysis conditions were 2 U/ml of α-L-rhamnoside at pH 5.5 and 85°C for 60 min. Overall, kae-3-O-rut could be completely hydrolyzed to kae by α-L-rhamnoside and β-glucosidase under the optimized conditions.

Kae-7-O-glu was also completely hydrolyzed by β-glucosidase under optimal conditions.

## Discussion

Kae showed the best antitumor, antioxidant and anti-inflammatory activities, whereas kae-3-O-rha, kae-7-O-glu and kae-3-O-rut showed poor activities. Kae had a stronger antiproliferation effect on HepG2 cells (IC_50_ = 30.92 μM), CT26 cells (IC_50_ = 88.02 μM) and B16F1 cells (IC_50_ = 70.67 μM). Antitumor activity differences could be linked to kae’s ability to significantly inhibit AKT phosphorylation and cleave caspase-9, caspase-7, caspase-3 and PARP. The participation of ROS in cancer systems also plays an important role. Using the DPPH and ABTS assay, we found that kae has the highest free radical scavenging activity, followed by kae-7-O-glu, while the other two compounds showed no significant activity. ROS has also been reported to participate in inflammation [34]. The antioxidant activity difference between kae and its glycosides indicated that kae may have better anti-inflammatory activity. To verify that, we studied the effect of these four compounds on T-cell proliferation and NO release from LPS-induced RAW 264.7 cells. Compared to the other compounds, kae significantly inhibited T-cell proliferation and NO release, which confirmed our hypothesis. Thus, kae aglycone has the strongest antitumor, antioxidant and anti-inflammation activities.

Kae mainly exists in the glycoside form in nature. Therefore, enzymatic hydrolysis of kae glycosides to produce kae was examined. We chose enzymes which had been previously used in our laboratory and optimize the hydrolysis conditions. Kae-7-O-glu and kae-3-O-rut could be completely hydrolyzed to kae under the optimal hydrolysis conditions, while kae-3-O-rha could not be hydrolyzed. Further studies are needed to identify a rhamnosidase that can efficiently hydrolyze kae-3-O-rha. This work presents a novel and efficient preparation of high-value flavone-related products.

## Supporting information

S1 Fig High-performance liquid chromatograms of kae-3-O-rut, kae-7-O-glu and their hydrolysis product kae. (A) Kae-3-O-rut standard. (B) The kae-3-O-rut hydrolysis product kae. (C) Kae-7-O-glu standard. (D) The kae-7-O-glu hydrolysis product kae.
(TIF)
