# Supplementary figures and images for: Antitumor, antioxidant and anti-inflammatory activities of kaempferol and its corresponding glycosides and the enzymatic preparation of kaempferol
Source: PLoS One. 2018 May 17;13(5):e0197563. doi: 10.1371/journal.pone.0197563 (PMC5957424; doi:10.1371/journal.pone.0197563)

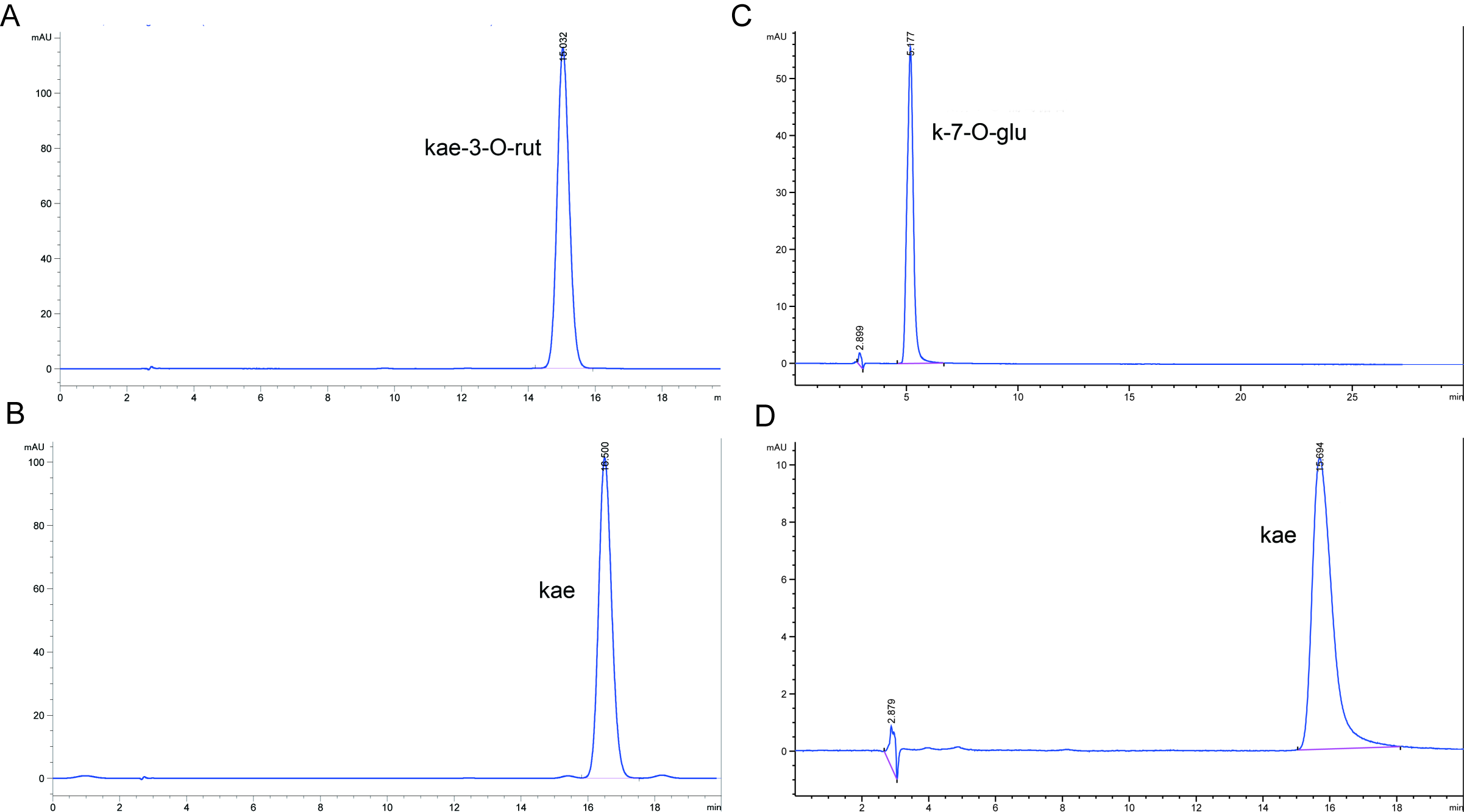

Supplement: S1 Fig — (A) Kae-3-O-rut standard. (B) The kae-3-O-rut hydrolysis product kae. (C) Kae-7-O-glu standard. (D) The kae-7-O-glu hydrolysis product kae. (TIF) [file pone.0197563.s001.tif]
